# A Literature Review on Care at the End-of-Life in the Emergency Department

**DOI:** 10.1155/2012/486516

**Published:** 2012-03-06

**Authors:** Roberto Forero, Geoff McDonnell, Blanca Gallego, Sally McCarthy, Mohammed Mohsin, Chris Shanley, Frank Formby, Ken Hillman

**Affiliations:** ^1^Simpson Centre for Health Services Research, South Western Sydney Clinical School (Liverpool Hospital) and The Australian Institute of Health Innovation (AIHI), University of New South Wales, Level 1, AGSM Building (G27), Kensington Campus, Gate 11, Botany Street, Randwick, NSW 2052, Australia; ^2^Centre for Health Informatics, The Australian Institute of Health Innovation (AIHI), University of New South Wales, Level 1, AGSM Building (G27), Kensington Campus, Gate 11, Botany Street, Randwick, NSW 2052, Australia; ^3^Emergency Care Institute, NSW Agency for Clinical Innovation, Tower A, Level 15, Zenith Centre, 821-843 Pacific Highway, Chatswood, NSW 2067, Australia; ^4^Australasian College for Emergency Medicine, 34 Jeffcott Street, West Melbourne, VIC 3003, Australia; ^5^Clinical Excellence Commission of New South Wales, Locked Bag A4062, Sydney South, NSW 1235, Australia; ^6^School of Public Health and Community Medicine, University of New South Wales, Level 3, Samuels Building, Randwick, NSW 2052, Australia; ^7^Aged Care Research Unit, South Western Sydney Clinical School, University of New South Wales, Liverpool Hospital, Level 2, 2-4 Speed Street, Liverpool, NSW 2170, Australia; ^8^Palliative Care Services, Illawarra Shoalhaven Local Health District, David Berry Hospital, 85 Tannery Road, Berry, NSW 2535, Australia; ^9^Centre for Clinical Governance Research in Health, Australian Institute of Health Innovation (AIHI), University of New South Wales, Level 1, AGSM Building (G27), Kensington Campus, Gate 11, Botany Street, Randwick, NSW 2052, Australia; ^10^Intensive Care Department, Liverpool Hospital, Liverpool, NSW 2170, Australia

## Abstract

The hospitalisation and management of patients at the end-of-life by emergency medical services is presenting a challenge to our society as the majority of people approaching death explicitly state that they want to die at home and the transition from acute care to palliation is difficult. In addition, the escalating costs of providing care at the end-of-life in acute hospitals are unsustainable. Hospitals in general and emergency departments in particular cannot always provide the best care for patients approaching end-of-life. The main objectives of this paper are to review the existing literature in order to assess the evidence for managing patients dying in the emergency department, and to identify areas of improvement such as supporting different models of care and evaluating those models with health services research. The paper identified six main areas where there is lack of research and/or suboptimal policy implementation. These include uncertainty of treatment in the emergency department; quality of life issues, costs, ethical and social issues, interaction between ED and other health services, and strategies for out of hospital care. The paper concludes with some areas for policy development and future research.

## 1. Introduction

Emergency departments (EDs) are increasingly used for patients, whether they are seriously ill as a result of being at the end-of-life (EOL) or whether they have a mainly acute and potentially treatable condition [1]. A growing number of patients at the EOL are admitted to EDs and receive increasingly invasive care [2, 3]. Clinical, social, and economic dimensions have been identified as key aspects of concern [4–14].

Studies exploring the impact of hospital care on the quality of the dying process highlight frequent, unsustainable, and sometimes unnecessary costs for an already stressed healthcare system [4, 5, 15–17].

There is also evidence that some patients at EOL experience a dying process that not always complies with the basic understanding of a “good death” [3, 18–23]. A comprehensive framework to improve care for the dying was developed by the Institute of Medicine (IOM): “*A decent or good death is the one that is: free from avoidable distress and suffering for patients, families, and caregivers; in general accord with patients' and families' wishes; and reasonably consistent with clinical, cultural, and ethical standards”. *The IOM framework also described several initiatives to improve the care of the dying, but there is limited knowledge about how these models of care for patients at EOL can be applied in the ED [18].

The main purpose of Emergency Medicine (EM) is to treat undifferentiated patients across age and disease or injury spectra, to create a time-restricted assessment of the patient, to resuscitate and stabilise in order to establish initial or definitive treatment, and finally to discharge the patient to an appropriate facility [24–32].

In the case of patients at EOL, these principles cannot always be applied or implemented because these patients cannot be treated in the same manner as patients with no terminal conditions [14, 33, 34]. Secondly, patients at EOL cannot be assessed and treated on the same time-restricted manner as other patients because they often present with multiple complications and several complex conditions [14, 35, 36]. Thirdly, resuscitation and active treatment for these patients may not be the best nor the preferred option, especially if those outcomes are against their wishes [37, 38], and they cannot always be discharged from ED to an appropriate facility or service [17, 39, 40].

Of the estimated 140,000 Australians who die each year [41], it is calculated that at least 100,000 of them die as a result of an “anticipated” [21] or “expected” death [42]. Of the 140,000 who die each year, 54% die in acute care hospitals; 20% die in hospices/palliative care; 16% die at home; 10% die in nursing homes [43]. The elderly tend to be overrepresented in EDs [44, 45].

The characteristics of patients at EOL comprise a wide range of demographic, clinical, and psychosocial factors in relation to time and place of death [21, 46–48]. They also comprise a wide range of ages [4, 11, 49–55] and conditions such as cancer [3, 4, 19, 24, 25, 56–62], terminal respiratory diseases [63–66], cardiac failure [26, 67–70], profound intellectual and physical disability, and advanced dementia [1, 47, 50, 62, 71, 72]. These patients pose a challenge in the ED because the majority appear not to have access to palliative care options, in particular those with non-cancer conditions. [73] Patients are managed in the ED when palliative management at home would have been more appropriate if a transition from active to palliative management had been made earlier [55, 74–76].

In the past, the limits of conventional medical treatment were largely determined by the general practitioner (GP). Today, it is more common for the GP to refer the patient to hospital when the illness becomes severe and life is threatened [2, 53, 77, 78]. The GP often faces barriers when managing their patients at EOL making their central role in home-based care difficult. Reasons include unexpected hospitalisation contrary to home death as planned as a result of the carer not having sufficient resources and an inability to control unpleasant symptoms. As a result the GP is often bypassed and the patient is admitted to an acute hospital where EDs tend to perform acute resuscitation and transfer to general wards or intensive care units (ICUs) [7, 24, 26, 53, 68, 70, 77, 79–85].

The following definitions of end of life, end-of-life care and palliative care, are used throughout this position paper.

- *End-of-life*. it is that part of life where a person is living with, and impaired by, an eventually fatal condition, even if the prognosis is ambiguous or unknown [42, 86].
- *End-of-life care*. EOL care combines the broad set of health and community services that care for the population at the end of their life. Quality end-of-life care is realised when strong networks exist between specialist palliative care providers, primary generalist providers, primary specialists, and support care providers and the community—working together to meet the needs of the people requiring care [86].
- Palliative care is specialist care provided for all people living with, and dying from an eventually fatal condition and for whom the primary goal is quality of life [87].

In this paper, we discuss the evidence around managing patients at the EOL in the ED setting, in particular some of the clinical, social, ethical, and economic aspects reported in the literature. We also define areas of research and policy development that have not been covered and suggest some potential courses of action.

## 2. Method of Review

We used MEDLINE and EMBASE databases and CareSearch palliative care filter for PubMed [88] to search for articles containing the key words described on Table 1. Articles were included if they described issues about “terminal care” or “end-of-life” and if they mentioned the “emergency department”; if they explored any “clinical”, “social”, “ethical”, or “economical” issues; or if they referenced “palliative care” or “intensive care”, “last year of life”, “death”, or “dying”. We also retrieved relevant grey literature on the topic such as strategic plans and unpublished commissioned research, editorials and policy documents, as well as instruments used in palliative and EOL care.

Of the 356 articles indicated on Table 1, 146 articles were rejected on the basis that they did not appear relevant after screening by title and abstract content. The remaining 210 articles were downloaded, printed, and reviewed for content and quality of evidence. Of these, 50 were excluded as they were not related to ED care. The remaining articles were then sorted using a descriptive thematic approach. We also used SCOPUS [89] to identity the number of citations associated with selected articles.

## 3. Results

Of the 160 papers included in this study, most had low citation levels when compared with articles from the same journal on different topics. Some examples included a paper about a good death published in *The Lancet* with 244 citations, but in relation to other topics, the top 100 most cited articles published by that journal, during the same period, had over 1200 citations each [18]. Another example is the case of two articles from a European study conducted across 174 EDs published by *Intensive Care Medicine *with 9 and 1 citations, respectively, while the top 100 most cited articles of this journal published in the same year had over 130 citations each [24, 25]. A further case is an Australian study auditing ED deaths published in 2009 by *Emergency Medicine Australasia. *This article had no citations while the top 50 articles of that journal on the same year of publication had over 10 citations each [90].

The same phenomena were noted across different journals regardless of their impact factor. The low citation rate can be explained partially by an expansion of the literature in recent years. Of the total documents in this review, 35% were published in 2010-2011; 33% were published in 2007–2009; 19% were published between 2000 and 2006; 13% were published between 1991 and 1999.

Overall, 6 articles (4%) had over 100 citations each [18, 19, 83, 91–93]; three articles (2%) had between 51 and 100 citations each [44, 94, 95]; 36 articles (22%) had between 10 and 50 citations each; 60 articles (37%) had between 1 and 9 citations each; 37 articles (23%) had no citations; 18 documents (12%) were not in Scopus or not applicable.

The thematic analysis identified six main topics of interest, namely, uncertainty of treatment in ED for patients at EOL, quality of life issues, costs, ethical and social issues, interaction between ED and other services, and strategies for out of hospital care.

### 3.1. Uncertainty of Treatment in the Emergency Department for Patients at EOL

O'Connor et al. indicated that in EDs, clinicians often play the role of commencing measures which might be judged to be futile in retrospect, and they argue that the ED environment could be an ideal place for difficult conversations, and difficult decisions regarding withholding of treatment [31]. They also indicated that treatment uncertainty is difficult to measure as it is usually the result of attempting to reach a consensus. Medical futility has been defined both quantitatively by the probability of the success of a treatment and qualitatively by the perceived value in terms of quality of life [31].

Several articles confirmed that this might be the case in a number of elderly patients with underlying chronic conditions where they may receive active treatment knowing that they will either die in the ED or be admitted by hospital staff knowing that they would die in the near future [24–32]. Reasons for these actions have been associated with miscommunications between doctors and patients; carers and patients, and between health care staff, and lack of information or cultural factors [29, 31, 39, 94, 96, 97].

Prognostication based on symptom characteristics such as recurrent infections, weight loss, falls, and functional decline has been used in predicting end-stage disease [60, 98]. Specific symptoms of certain conditions such as ovarian carcinoma have specifically been associated with shorter survival times, namely, bone marrow depression, renal failure, and dyspnoea [99–101]. Validation studies related to basic life support have been conducted to identify patients with no probability of survival. One study found that following rules for the termination of basic life support had a positive predictive value for death of 99.5% and decreased the transfer of patients to hospital by 63% [68].

The review also found that the reliability of prognostic models by critical care specialists and ED physicians regarding impending mortality of patients with poor functional outcome is not consistent [17]. In a six-month follow-up study, the reliability of prognosis had a low sensitivity of 35–37% suggesting that only one third of people with poor prognosis were likely to die. In addition, they had a very high specificity of 99% and positive predictive values of 96%, suggesting that most people expected to live survived (only 1% die when they were expected to live) [29, 31, 96, 102].

In summary, this review found that clinicians in the ED confront a major dilemma about uncertainty of treatment. The dilemma is whether an accurate prediction of appropriate active treatment is possible and whether decisions about a transition to palliation can be made [60, 96].

### 3.2. Quality of Life Issues

Optimising the quality of life of those with chronic life limiting illness is an important challenge. The International Council of Nursing (ICN) mandates that “nurses have a unique and primary responsibility for ensuring that individuals at the EOL experience a peaceful death” [36]. As indicated before, the literature supports the notion that most deaths of patients at EOL do not comply with good death principles [14, 18, 73].

Wright et al. [3], demonstrated that patients with cancer who die in hospital ward or ICU are more likely to have worse quality of life compared with those who die at home, and their bereaved caregivers were more likely to develop psychiatric illness. They concluded that interventions aimed at decreasing terminal hospitalisations or increasing hospice utilisation may enhance patients' quality of life at the EOL and minimise bereavement-related distress.

In relation to tools to improve quality of life, the literature has shown several strategies to inform patients' wishes, choices, and treatment options as illness progresses and death approaches such as advanced care planning and other tools [1, 7, 21, 48, 74, 77, 103, 104]. These comprise models of care pathways and tools to facilitate the transition from active to palliative care [1, 7, 21, 48, 67, 103–108].

The best known is the Liverpool Care Pathway (LCP) [57, 109]. It focuses on three main areas of care: communication between staff about knowledge of the patient and their family's wishes, improving the consistency of care while dying and after death, and improving the perceived quality of care given to the family.

It has been reported that the real challenge for ED physicians is to be certain of the appropriateness of placing patients on such pathways [52, 57, 67, 110]. A modified version of this instrument known as the Modified LCP was implemented and validated in the ED in the UK, to investigate its applicability in the ED environment [52]. The authors found that it was possible to make it more relevant for the ED by shortening the pathway and administering medication in a way familiar to ED staff [52, 67].

There is also evidence that the skills required to manage patients with terminal conditions in the ED are different to those required to resuscitate and stabilise nonterminal ED patients [31, 36, 111]. Another factor reported in the literature affecting quality of life at the EOL is the increasing tendency to specialise in medicine [36, 39, 112]. The principles of traditional medical practice such as concentrating on optimising the function of the organ or area have been reported as inappropriate in most situations of EOL care [2, 23, 76, 113, 114].

### 3.3. Costs

The International Council of Nursing (ICN) estimates that more than 1000 million people by 2020 will be over the age of 60, and three quarters of all deaths will be caused by noncommunicable diseases worldwide [36]. Health expenditure in the last years of life has confirmed that increased costs are associated with proximity to death. Many articles have confirmed that up to half of all medical costs are in the last 6–12 months of life [1, 4, 5, 36, 48, 51, 58, 73, 92, 115].

In a longitudinal study conducted across 59 nursing homes in Maryland, United States, between 1992 and 1995; it was found that residents who died during the 2-year study period had significantly greater mean rates of utilisation of all types of health care than residents who were not discharged from the nursing home, even when controlling for dementia, age, functional status, and number of comorbid conditions. Those who died within a month of admission had significantly more ED and medical visits than those who died after a longer stay. They concluded that the pattern of high healthcare utilisation before death was consistent with studies of the overall Medicare population that show an increase in Medicare expenditures in the period before death [17].

Unwanted and unnecessary hospitalisation is a contentious issue that is common amongst patients at EOL [36, 56, 79, 116–118]. Several papers have demonstrated that symptom control is often a legitimate reason for acute hospital admission [23, 36, 46, 48, 96, 103]. Acute problems in cancer patients are sepsis, respiratory obstruction, spinal cord compression, metabolic disturbances, gastrointestinal tract obstruction, and deep vein thrombosis [52]. It has been reported that there is a difference between symptoms which are treated without reference to their cause and acute problems which need to be identified and then treated by the usual medical means [83].

However, hospitalisation involves expensive decisions; it has been estimated that average chemotherapy costs are approximately US$ 8,000 per month; an ICU bed costs almost US$4,000 per day; standard surgery costs US$5,000 per hour [72]. It was estimated in 2002 that in the US alone 33% of those who die in hospital spent an average of 10 days in ICU and 80% preferred to die at home [36, 56, 116–118]. In general, between 45% and 70% of patients at EOL want to die at home but up to 70% die in acute hospitals [3, 46, 70, 79, 83].

Family caregivers currently provide a high proportion of home-based care at EOL; however, there is little information about estimating the full costs incurred by patients and their caregivers. Most economic analyses of home-based care are limited to measurement of publicly financed care [58, 119]. Despite the fact that a high proportion of care at EOL is provided by family caregivers, particularly in the home setting, time spent by these caregivers is often perceived as having no or minimal monetary value [42, 104, 119, 120]. In this context, care costs at EOL can be greatly underestimated. A framework including long-term as well as cost-effective alternatives is required from a societal perspective [4, 5, 104, 119].

### 3.4. Ethical and Social Issues

Ethical issues around EOL care have been recognised by emergency physicians [81, 121, 122] and paramedics [70] in many different settings in relation to cardiopulmonary resuscitation (CPR), palliative care programs and advance care directives (ACDs) [31, 47, 48, 53]. Euthanasia and the right to die have also been explored, in particular those aspects associated with dignified death, legislated resuscitation [70, 123], attitudes to death and the illusion of autonomy at the EOL [13, 123–126]. In this context, illusion of autonomy is understood as the decision of physicians to ignore patient preferences about life-sustaining care. Some research has also reported that health care professionals are able to make correct estimations of patients' preferences but they are not always aware of their own bias [127].

There have been discussions about palliative care and pain control as a human right, as well as some legislated requirements for palliative care [72, 83, 126]. In relation to religion and spirituality, the ED environment provides limited privacy. It may not be conducive to enhancing the respect and comfort required by the grieving family and may limit communication with the family. The training of staff on spiritual issues is often inadequate and the role of chaplains, clerics, ethnic health workers, interpreters, and social workers may not be considered essential in the ED environment. The LCP has an item for calling a priest; however it is not always covered as LCP is basically a checklist which in principle should improve care of the immediately dying by making sure that the basics are covered but evidence-based information is limited [6, 10, 32, 128–133].

### 3.5. Interaction between ED and Other Health Services

The review has also found several articles describing the relationship between EDs, hospital services such as ICUs, hospital wards, ambulance services, and other community-based health services such as residential care facilities and GPs [12, 35, 39, 42, 48, 53, 113]. Most articles have indicated important problems in relation to communication and degree of preparedness for the death [6, 74, 83, 132, 134]. These include conflicts that may arise when there is pressure by treating clinicians and relatives to admit patients into ICUs, and unreal expectations of relatives or friends of patients clashing with the reality of dying and inappropriateness of further treatment [39, 55, 74].

To alleviate these problems, several alternatives have been proposed such as physicians' orders and ACDs. These provide alternatives about life-sustaining treatments in the ED, especially for patients who are terminally ill and for those requiring treatment by emergency physicians who may not be familiar with the background to the patients' illness [10, 37, 49, 53, 60, 95, 103, 135]. The International Society of Advance Care Planning and End of Life Care (ACPEL) has been formed recently to coordinate and promote alternative plans for patients at EOL [136]. These plans would be advantageous to physicians in the ED if such patients had an ACD, preferably a combined proxy-treatment directive, and preferably one that has been thoroughly discussed with the family physician and with the proxy, successor proxies, and preferably relatives and friends [75, 76].

In relation to ambulance services, there have been examples where State laws in the US allow paramedics to follow Physician Orders for Life Sustaining Treatment (POLST) or Medical Orders for Life Sustaining Treatment (MOLST) forms limiting life-sustaining treatments such as CPR and intubation [7, 10, 39, 45, 72, 75]. Before admission to the ED, paramedics may experience conflict with terminally ill patients who would rather be managed at home. Paramedics are frequently called to attend terminally ill patients after a cardiac arrest [70]. Situations such as this make management decisions in the ED difficult. Current regulations are a source of conflict between the paramedic's duty to treat and the patient's right to limit resuscitative efforts at the time of death [70, 137–139].

In the area of profound intellectual disability, there is often a need for chronic palliative care, with a formal documented and agreed decision that the goal of treatment is to “allow natural death” and not to continue with life prolonging procedures such as long-term percutaneous endoscopic gastrostomy feeding tubes. Who and when to make this decision can be a problem and communicating it in a binding way to those who may become involved in further health care can be complex [50, 71, 140]. The existence of ACDs should ideally be on the electronic medical record as an “alert”. However, relatives also need to have the ACD to show to staff unfamiliar with the patient.

Alternative palliative care services are needed in order to avoid ED admissions, including home-based pain relief and intensive nursing care and carer support. Caregivers need to know what to expect at the EOL [116]. For many caregivers, dealing with a terminally ill patient is their first experience in witnessing an impending death, and often they are not given appropriate education or support [12, 116]. As a consequence, many of the normal body responses are interpreted as medical emergencies and result in unnecessary emergency room visits and frantic calls to health care providers [12, 78]. Community nurses in Australia usually provide these services, but in the ED, they are not available. For that reason, in the ED, counsellors or trained palliative care nurses and physicians may be helpful in ensuring that caregivers and their patients are aware of signs such as reduced appetite and cognitive dysfunction [3, 12, 73, 78, 95, 104, 128, 130, 135, 141–145].

### 3.6. Strategies for out of Hospital Care

Since the SUPPORT study in 1995, there have been landmark improvements around palliative care support for people with terminal conditions [83, 146]. Unfortunately these conditions are not reaching many people in EDs [31, 33].

The paradox is that while there is strong evidence suggesting that the majority of such patients do not want to die either in the ED, ICU or hospital ward, there is very limited information about strategies for out of hospital care in the ED [3, 70, 79, 142, 147, 148].

In general, between 45% and 70% of people have been reported to prefer dying at home, but between 60% and 86% die in acute hospitals [3, 36, 46, 70, 79, 83]. In the US alone 80% of those who die in hospital prefer to die at home. The reality is that most terminally ill patients are managed in institutions [4, 149, 150].

Several articles have reported that when patients are offered a choice between active management and active management together with out of hospital care, they would often choose the latter. Those patients tended to live longer and had a better quality of life [1, 3, 23, 62, 91, 93, 114].

In the case of GPs, it has been reported that hospitalisation can be avoided if home care for the dying was better supported with access to experts in palliation and improved communication within the health service [53, 77, 79].

A French medical emergency call centre responded to requests for emergency aid at the homes of terminally ill patients by sending to the patient's home a nurse and a volunteer, both trained in hospice (terminal) care, together with a physician and the emergency ambulance team. When the patient wished to stay at home, the hospice team remained to support the patient and family and to provide comfort care until the crisis situation. This type of organisation was consistent with respect for the patient's choice to remain at home until death. Prevention of unwanted hospitalisation can result in cost savings which can, in turn, be used to fund such a program [117, 151].

Do Not Resuscitate (DNR) orders and ACDs have been considered by family physicians and other medical professionals. However, it has been reported that ACDs have no legal force in some Australian states, and relatives can override them, just like with organ donation [70, 83, 152–155].

In relation to hospice care, in the United States, the term “hospice” generally refers to in-home care, hospices are not institutions, and hospice care is usually provided by nurses or volunteers with no specialist palliative care training or without the support of medical staff and often with no medical staff at all [8]. In some settings, approximately 50% of terminally ill patients receive hospice home care services before death but the total population level is unknown [56, 102].

The literature has also identified some determinants of hospice home care use among terminally ill cancer patients including longer length of survival, perceived greater family ability to achieve preferred place of death, home as the realistic preferred place of death, being female, lower levels of functional dependency, and use of emergency care during the final days of life. Some authors have even indicated that it is often underutilised, despite the widespread availability of hospice services [56]. Sometimes terminally ill cancer patients who may benefit from hospice care do not receive it, even when alternative care is available.

## 4. Summary and Conclusions

As patients approach EOL, their disease process may create an immediate life-threatening emergency normally requiring admission to an ED, yet further active treatment is futile. A large number of elderly patients at EOL die in the ED and many of them are admitted to hospital knowing that they are dying and that modern interventional medicine has little to offer [10, 24–26, 29, 30, 154].

As indicated at the beginning, the main purpose of EM is to treat undifferentiated patients across age and disease or injury spectra, to create a time-restricted assessment of the patient, to resuscitate and stabilise in order to establish initial or definitive treatment, and finally to discharge the patient to an appropriate facility [24–32]. As unravelled in this paper, these principles are not always possible to implement exactly as prescribed when managing patients facing EOL. Organising the most appropriate care for these patients can be time-consuming, requiring honest and timely prognostic information, clear recommendations, facilitating patient-family discussions, and affirming patient choices [154].

Following is a list of policy and practice recommendations and discussion of the need for further research derived from the findings of this review.

### 4.1. Better Prognostic Models

The reliability of estimating death for patients with poor functional outcomes is low [52, 57, 67, 102, 110]. Treatment futility is also difficult to prognosticate and is complicated by variation of what may be perceived as an acceptable probability of success but an unacceptable level of quality of life or vice versa [31, 96]. More precise instruments with higher sensitivity to assess EOL timelines in the ED are needed. That knowledge could be used to offer a timely transition from active treatment to palliative care with the suspension of futile care in the ED.

### 4.2. Specialised Training

Knowing when to shift from life-prolonging treatment to palliative approaches, and focusing on quality of life and comfort, is emotionally challenging for patients, families, and physicians. Most practitioners in emergency medicine are not trained to recognise and manage patients at the EOL. Increased training among ED clinicians in communication about goals of care and EOL care is therefore required urgently. Simulation training can be used to improve EOL management skills of ED clinicians.

### 4.3. A Structured Approach to Decision Making

Most elderly patients have multiple problems and comorbidities resulting in “committee” medicine with little leadership or coordination to initiate discussions about the value of continuing conventional medicine and changing the care to support the dying process [2, 39, 113, 156]. A structured approach to decision making with clear steps similar to an LCP but adapted to ED is needed. It should include prognosis; risk-benefit analysis of proposed interventions taking into account current symptom burden as well as patient's age, life stage, and goals of care; exploring and arranging appropriate palliative care if needed; making clear recommendations; and taking steps to ensure that appropriate support is provided to patients and their families.

### 4.4. Infrastructure, Information, and Planning Ahead

Lack of information and planning for a home death are associated with the absence of action plans for anticipated symptoms and deteriorations and the inability to alter management at short notice and the underrecognition that a patient is “palliative” and active ED care for him or her might not be appropriate. These issues have been identified in multiple policy documents [1, 15, 16, 21, 41–43, 46–48, 73, 86, 110, 112]. Equally important is creating sustainable strategies for out of hospital care [45, 46, 51, 62, 91, 116, 157–162].

### 4.5. Evidence-Based Research about EOL Care in the ED

There are few studies exploring interventions at the EOL across different settings, including the ED [24, 25, 83]. As indicated in the results, most of the studies included in this paper have received less citation than other topics when compared across journals. This was consistent across journals of different impact factors. That suggests that EOL research has received less attention amongst researchers and practitioners relative to other topics published in the same journals. In general we found that the level of citation in this area of research is comparatively low. Only 28% of the articles had more than 10 citations each and 60% had less than 9 citations each.

This can be partially explained by an expansion of the literature in recent years given that 35% of the articles were published in the last 24 months. However, in spite of this reason, the low citation rate is a cause for concern; EOL research might still not be receiving the appropriate attention it requires. Sixty-two percent of the articles were published after 2000 and 13% were published between 1991 and 1999. Only the SUPPORT study has received a high number of citations (1704) since 1995 [83]. Other 5 studies have also good citation levels [18, 19, 83, 91–93] but the large majority have had low citation numbers.

Furthermore, the patterns of life, disease, dying, and death have changed dramatically in recent years across the world [1, 21, 47, 74], and research is essential to develop evidence-based public health policy. Further research should include evaluation of pathways for the appropriate care of terminally patients in the ED (such as the LCP), comparison of services across countries such as the UK where GPs are required to do home visits on sick people and are financially penalised for hospital admissions and Australia, where this is not the case. Finally, we support the identification of robust rules for patients receiving inappropriate advanced life support care.

## Figures and Tables

**Table 1:** Terms used in the literature review (updated August 2011).

Strategy	MeSH (Medical Subject Headings)	Results
1	“Terminally ill”	7485
2	“Terminal care/or hospice care/or resuscitation orders/or withholding treatment”	30633
3	Attitude to Death/or Terminally Ill/or place of death	16612
4	1 and 2 and 3	2355
5	emergency or Emergencies	172112
6	4 and 5	92
7	Replicated steps 1–6 using EMBASE and PubMed/CareSearch	356

## References

[2] Hillman K (2010). Dying safely. *International Journal for Quality in Health Care*.

[3] Wright AA, Keating NL, Balboni TA, Matulonis UA, Block SD, Prigerson HG (2010). Place of death: correlations with quality of life of patients with cancer and predictors of bereaved caregivers' mental health. *Journal of Clinical Oncology*.

[4] Kardamanidis K, Lim K, Da Cunha C, Taylor LK, Jorm LR (2007). Hospital costs of older people in New South Wales in the last year of life. *Medical Journal of Australia*.

[5] Moorin RE, Holman CDJ (2008). The cost of in-patient care in Western Australia in the last years of life: a population-based data linkage study. *Health Policy*.

[6] Billings JA, Kolton E (1999). Family satisfaction and bereavement care following death in the hospital. *Journal of Palliative Medicine*.

[7] Billings JA, Krakauer EL (2011). On patient autonomy and physician responsibility in end-of-life care. *Archives of Internal Medicine*.

[8] Choi YS, Billings JA (2002). Changing perspectives on palliative care. *Oncology*.

[9] Kerrouault E, Denis N, Le Conte P, Dabouis G (2007). Improving organization ofcare could reduce referrals ofcancer patients totheemergency department. Prospective analysis of 123 patients. *Presse Medicale*.

[10] Miller RB (2009). Physician orders to supplement advance directives: rescuing patient autonomy. *Journal of Clinical Ethics*.

[11] Roch A, Wiramus S, Pauly V (2011). Long-term outcome in medical patients aged 80 or over following admission to an intensive care unit. *Critical Care*.

[12] Russell C, Middleton H, Shanley C (2008). Dying with dementia: the views of family caregivers about quality of life. *Australasian Journal on Ageing*.

[13] Virik K, Glare P (2002). Requests for euthanasia made to a tertiary referral teaching hospital in Sydney, Australia in the year 2000. *Supportive Care in Cancer*.

[14] Chan GK (2005). Understanding end-of-life caring practices in the emergency department: developing Merleau-Ponty’s notions of intentional arc and maximum grip through praxis and phronesis. *Nursing Philosophy*.

[15] Access Economics (2001). *Population Ageing and the Economy*.

[16] Australian Health Ministers (2010). *National Palliative Care Strategy 2010*.

[17] Bercovitz A, Gruber-Baldini AL, Burton LC, Hebel JR (2005). Healthcare utilization of nursing home residents: comparison between decedents and survivors. *Journal of the American Geriatrics Society*.

[18] Emanuel EJ, Emanuel LL (1998). The promise of a good death. *Lancet*.

[19] Glare P, Virik K, Jones M (2003). A systematic review of physicians’ survival predictions in terminally ill cancer patients. *British Medical Journal*.

[20] Leichtentritt RD, Rettig KD (2000). The good death: reaching an inductive understanding. *Omega*.

[21] National Palliative Care Strategy (2010). *Supporting Australians to Live Well at the End of Life*.

[22] Hales S, Zimmermann C, Rodin G (2008). The quality of dying and death. *Archives of Internal Medicine*.

[23] Institute of Medicine (1998). *Approaching Death: Improving Care at the End of Life*.

[24] Conte PL, Baron D, Trewick D (2004). Withholding and withdrawing life-support therapy in an Emergency Department: prospective survey. *Intensive Care Medicine*.

[25] Le Conte P, Riochet D, Batard E (2010). Death in emergency departments: a multicenter cross-sectional survey with analysis of withholding and withdrawing life support. *Intensive Care Medicine*.

[26] Van Walraven C, Forster AJ, Parish DC (2001). Validation of a clinical decision aid to discontinue in-hospital cardiac arrest resuscitations. *Journal of the American Medical Association*.

[27] Girbes ARJ (2004). Dying at the end of your life. *Intensive Care Medicine*.

[28] McCarthy S The Challenges in Australian Emergency Departments. http://www.digitalhospitaldesign.org/?q=node/3.

[29] Marco CA, Larkin GL, Moskop JC, Derse AR (2000). Determination of “futility” in emergency medicine. *Annals of Emergency Medicine*.

[30] Marco CA, Larkin GL (2000). Ethics seminars: case studies in “futility”—challenges for academic emergency medicine. *Academic Emergency Medicine*.

[31] O'Connor AE, Winch S, Lukin W, Parker M (2011). Emergency medicine and futile care: taking the road less travelled. *Emergency Medicine Australasia*.

[32] Cooke MW, Cooke HM, Glucksman EE (1992). Management of sudden bereavement in the accident and emergency department. *British Medical Journal*.

[33] Chan GK (2004). End-of-life models and emergency department care. *Academic Emergency Medicine*.

[34] Chan GK (2006). End-of-life and palliative care in the emergency department: a call for research, education, policy and improved practice in this frontier area. *Journal of Emergency Nursing*.

[35] Kompanje EJO (2010). The worst is yet to come. Many elderly patients with chronic terminal illnesses will eventually die in the emergency department. *Intensive Care Medicine*.

[36] McClain K, Perkins P (2002). Terminally ill patients in the emergency department: a practical overview of end-of-life issues. *Journal of Emergency Nursing*.

[37] Temel JS, McCannon J, Greer JA (2008). Aggressiveness of care in a prospective cohort of patients with advanced NSCLC. *Cancer*.

[38] Travis SS, Bernard M, Dixon S, McAuley WJ, Loving G, McClanahan L (2002). Obstacles to palliation and end-of-life care in a long-term care facility. *Gerontologist*.

[39] Hillman K, Chen J, Vincent J-L (2009). Managing conflict at the end-of-life. *Yearbook of Intensive Care and Emergency Medicine*.

[40] Gardiner C, Cobb M, Gott M, Ingleton C (2011). Barriers to providing palliative care for older people in acute hospitals. *Age and Ageing*.

[41] Australian Bureau of Statistics (2010). *3302.0-Deaths, Australia 2009*.

[42] Palliative Care Australia (2008). *Carers and End of Life-Position Statement*.

[43] Palliative Care Knowledge Network http://www.caresearch.com.au/caresearch/WhatisPalliativeCare/UnderstandingPalliativeCare/PreferredPlaceofDeath/tabid/407/mid/864/dnnprintmode/true/Default.aspx?SkinSrc=%5bG%5dSkins%2f_default%2fNo+Skin&ContainerSrc=%5bG%5dContainers%2f_default%2fNo+Container.

[44] Caplan GA, Brown A, Croker WD, Doolan J (1998). Risk of admission within 4 weeks of discharge of elderly patients from the emergency department—the DEED study. *Age and Ageing*.

[45] Fromme EK, Bascom PB, Smith MD (2006). Survival, mortality, and location of death for patients seen by a hospital-based palliative care team. *Journal of Palliative Medicine*.

[46] National Audit Office (2008). *End of Life Care: Report by the Comptroller and Auditor General*.

[47] NSW Health (2011). *Quality Care at the End of Life: Strategic Framework and Implementation Plan*.

[48] NSW Health (2011). *Advance Planning for Quality Care at End of Life*.

[49] Bezzina AJ (2009). Prevalence of advance care directives in aged care facilities of the Northern Illawarra. *Emergency Medicine Australasia*.

[50] Lee L, MacPherson M (2010). Long-term percutaneous endoscopic gastrostomy feeding in young adults with multiple disabilities. *Internal Medicine Journal*.

[51] Moorin RE, Holman CDJ (2007). Modelling changes in the determinants of PHI utilisation in Western Australia across five health care policy eras between 1981 and 2001. *Health Policy*.

[52] Paterson BC, Duncan R, Conway R, Paterson FM, Napier P, Raitt M (2009). Introduction of the Liverpool Care Pathway for end of life care to emergency medicine. *Emergency Medicine Journal*.

[53] Rhee JJ, Zwar NA, Kemp LA (2011). How is advance care planning conceptualised in Australia? Findings from key informant interviews. *Australian Health Review*.

[54] Rodriguez KL, Hanlon JT, Perera S, Jaffe EJ, Sevick MA (2010). A cross-sectional analysis of the prevalence of undertreatment of nonpain symptoms and factors associated with undertreatment in older nursing home hospice/palliative care patients. *The American Journal of Geriatric Pharmacotherapy*.

[55] Shanley C, Whitmore E, Khoo A, Cartwright C, Walker A, Cumming RG (2009). Understanding how advance care planning is approached in the residential aged care setting: a continuum model of practice as an explanatory device. *Australasian Journal on Ageing*.

[56] Tang ST (2003). Determinants of hospice home care use among terminally ill cancer patients. *Nursing research*.

[57] Costantini M, Ottonelli S, Canavacci L (2011). The effectiveness of the Liverpool care pathway in improving end of life care for dying cancer patients in hospital. A cluster randomised trial. *BMC Health Services Research*.

[58] Moorin RE, Holman CDJ (2006). The effects of socioeconomic status, accessibility to services and patient type on hospital use in Western Australia: a retrospective cohort study of patients with homogenous health status. *BMC Health Services Research*.

[59] Clift E (2011). Hospice and the “End Game”. *Health Affairs*.

[60] Glare P, Sinclair C, Downing M, Stone P, Maltoni M, Vigano A (2008). Predicting survival in patients with advanced disease. *European Journal of Cancer*.

[61] Goodman D, Bronner K (2010). Quality of end-of-life cancer care for medicare beneficiaries. Regional and hospital specific analysis. *A Report of the Dartmouth Atlas Project*.

[62] Mccarron M, Mccallion P, Fahey-Mccarthy E, Connaire K (2011). The role and timing of palliative care in supporting persons with intellectual disability and advanced dementia. *Journal of Applied Research in Intellectual Disabilities*.

[63] Small N, Gardiner C, Barnes S (2010). Using a prediction of death in the next 12 months as a prompt for referral to palliative care acts to the detriment of patients with heart failure and chronic obstructive pulmonary disease. *Palliative Medicine*.

[64] Gardiner C, Barnes S, Small N (2010). Reconciling informed consent and do no harm: ethical challenges in palliative-care research and practice in chronic obstructive pulmonary disease. *Palliative Medicine*.

[65] Gardiner C, Gott M, Small N (2009). Living with advanced chronic obstructive pulmonary disease: patients concerns regarding death and dying. *Palliative Medicine*.

[66] Gott M, Gardiner C, Small N (2009). Barriers to advance care planning in chronic obstructive pulmonary disease. *Palliative Medicine*.

[67] Duncan RA, Paterson B (2008). Can we improve the care of patients dying in the emergency department? nursing attitudes towards the intoruction of a Modified Liverpool Care Pathway. *Annals of Emergency Medicine*.

[68] Sherbino J, Keim SM, Davis DP (2010). Clinical decision rules for termination of resuscitation in out-of-hospital cardiac arrest. *Journal of Emergency Medicine*.

[69] Grech C, Pannell D, Smith-Sparrow T (2001). The delay in transfer between the emergency department and the critical care unit for patients with an acute cardiac event—in hospital factors. *Australian Critical Care*.

[70] Guru V, Verbeek PR, Morrison LJ (1999). Response of paramedics to terminally ill patients with cardiac arrest: an ethical dilemma. *Canadian Medical Association Journal*.

[71] McCarron M, McCallion P (2007). End-of-life care challenges for persons with intellectual disability and dementia: making decisions about tube feeding. *Intellectual and Developmental Disabilities*.

[72] Fromme EK, Guthrie AE, Grueber CM (2011). Transitions in end-of-life care: the Oregon trail. *Frontiers of Health Services Management*.

[73] Economist Intelligence Unit (2010). *The Quality of Death. Ranking End-of-Life Care Across the World*.

[74] Shanley C, Sutherland S, Tumeth R, Stott K, Whitmore E (2009). Caring for the older person in the emergency department. The ASET Program and the Role of the ASET Clinical Nurse Consultant in South Western Sydney, Australia. *Journal of Emergency Nursing*.

[75] Silvester W, Detering K (2011). Advance directives, perioperative care and end-of-life planning. *Best Practice and Research*.

[76] Silvester W, Detering K (2011). Advance care planning and end-of-life care. *Medical Journal of Australia*.

[77] Rhee JJO, Zwar N, Vagholkar S, Dennis S, Broadbent AM, Mitchell G (2008). Attitudes and barriers to involvement in palliative care by Australian urban general practitioners. *Journal of Palliative Medicine*.

[78] Kutzen H (1998). Caregivers need to know what to expect at the end of life. *Faculty Notes*.

[79] Abom BM, Obling NJ, Rasmussen H, Kragstrup J (2000). Unplanned emergency admission of dying patients. Causes elucidated by focus group interviews with general practitioners. *Ugeskrift for Laeger*.

[80] Ingarfield SL, Finn JC, Jacobs IG (2009). Use of emergency departments by older people from residential care: a population based study. *Age and Ageing*.

[81] Derse AR (2001). End-of-life care ethics. The emergency physician and end-of-life care. *The Virtual Mentor*.

[82] Downar J, Hawryluck L (2010). What should we say when discussing “code status” and life support with a patient? A Delphi analysis. *Journal of Palliative Medicine*.

[83] Connors AF, Dawson NV, Desbiens NA (1995). A controlled trial to improve care for seriously ill hospitalized patients. *Journal of the American Medical Association*.

[84] Wunsch H, Harrison DA, Harvey S, Rowan K (2005). End-of-life decisions: a cohort study of the withdrawal of all active treatment in intensive care units in the United Kingdom. *Intensive Care Medicine*.

[85] Bion J (2008). Patient safety: needs and initiatives. *Indian Journal of Critical Care Medicine*.

[86] Palliative Care Australia (2008). *Palliative and End of Life Care—Glossary of Terms*.

[87] World Health Organisation http://www.who.int/cancer/palliative/definition/en/#.

[88] CareSearch http://www.caresearch.com.au/caresearch/FindingEvidence/PubMedTopicSearches/tabid/322/Default.aspx.

[89] http://www.info.sciverse.com/scopus/.

[90] Clunas S, Whitaker R, Ritchie N, Upton J, Isbister GK (2009). Reviewing deaths in the emergency department: deaths in the department or deaths within 48 h. *Emergency Medicine Australasia*.

[91] Gomes B, Higginson IJ (2006). Factors influencing death at home in terminally ill patients with cancer: systematic review. *British Medical Journal*.

[92] Hitiris T, Posnett J (1992). The determinants and effects of health expenditure in developed countries. *Journal of Health Economics*.

[93] Temel JS, Greer JA, Muzikansky A (2010). Early palliative care for patients with metastatic non-small-cell lung cancer. *New England Journal of Medicine*.

[94] Campbell ML, Bizek KS, Thill M (1999). Patient responses during rapid terminal weaning from mechanical ventilation: a prospective study. *Critical Care Medicine*.

[95] Parker SM, Clayton JM, Hancock K (2007). A systematic review of prognostic/end-of-life communication with adults in the advanced stages of a life-limiting illness: patient/caregiver preferences for the content, style, and timing of information. *Journal of Pain and Symptom Management*.

[96] Le BHC, Watt JN (2010). Care of the dying in Australia’s busiest hospital: benefits of palliative care consultation and methods to enhance access. *Journal of Palliative Medicine*.

[97] Johnson KS, Kuchibhatla M, Tanis D, Tulsky JA (2008). Racial differences in hospice revocation to pursue aggressive care. *Archives of Internal Medicine*.

[98] Fromme EK, Smith MD, Bascom PB, Kenworthy-Heinige T, Lyons KS, Tolle SW (2010). Incorporating routine survival prediction in a U.S. hospital-based palliative care service. *Journal of Palliative Medicine*.

[99] Sun XG, Wu M, Ma SQ, Li CY, Jin LN, Shen K (2007). Study of symptoms in terminally ill patients with ovarian carcinoma. *Chinese Journal of Obstetrics & Gynecology*.

[100] Travis SS, Moore S, Larsen PD, Turner M (2005). Clinical indicators of treatment futility and imminent terminal decline as discussed by multidisciplinary teams in long-term care. *American Journal of Hospice and Palliative Medicine*.

[101] Van Der Steen JT, Helton MR, Ribbe MW (2009). Prognosis is important in decisionmaking in Dutch nursing home patients with dementia and pneumonia. *International Journal of Geriatric Psychiatry*.

[102] Rodriguez RM, Wang NE, Pearl RG (1997). Prediction of poor outcome of intensive care unit patients admitted from the emergency department. *Critical Care Medicine*.

[103] Byrne M, Tresgallo M, Saroyan J, Granowetter L, Valoy G, Schechter W (2011). Qualitative analysis of consults by a pediatric advanced care team during its first year of service. *American Journal of Hospice and Palliative Medicine*.

[104] Guerriere DN, Zagorski B, Fassbender K, Masucci L, Librach L, Coyte PC (2010). Cost variations in ambulatory and home-based palliative care. *Palliative Medicine*.

[105] McGinn M (2010). End of life care. *European Journal of Cancer Care*.

[106] Luhrs CA, Penrod JD (2007). End-of-life care pathways. *Current Opinion in Supportive and Palliative Care*.

[107] Hardy JR, Haberecht J, Maresco-Pennisi D, Yates P (2007). Audit of the care of the dying in a network of hospitals and institutions in Queensland. *Internal Medicine Journal*.

[108] Mellor F, Foley T, Connolly M, Mercer V, Spanswick M (2004). Role of a clinical facilitator in introducing an integrated care pathway for the care of the dying. *International Journal of Palliative Nursing*.

[109] Veerbeek L, van Zuylen L, Swart SJ (2008). The effect of the Liverpool Care Pathway for the dying: a multi-centre study. *Palliative Medicine*.

[110] Department of Health, W.A., WA Cancer and Palliative Care Health Network (2009). *A pilot study of the use of the Liverpool Care Pathway in Western Australia. Final Report*.

[111] Council GM, General Medical Council (2010). Treatment and care towards the end of life: good practice in decision making. *Guidance to Doctors*.

[112] Australian Medical Council Good Medical Practice: A Code of Conduct for Doctors in Australia. http://www.amc.org.au/index.php/about/good-medical-practice.

[113] Hillman KM (2011). End-of-life care in acute hospitals. *Australian Health Review*.

[114] Akiyama A, Hanabusa H, Mikami H (2011). Factors influencing home death in Japanese metropolitan region. *Journal of Ageing Research*.

[115] Felder S, Meier M, Schmitt H (2000). Health care expenditure in the last months of life. *Journal of Health Economics*.

[116] Fromme EK, Tilden VP, Drach LL, Tolle SW (2004). Increased family reports of pain or distress in dying Oregonians: 1996 to 2002. *Journal of Palliative Medicine*.

[117] Ciais JF, Pradier C, Ciais C, Berthier F, Vallageas M, Raucoules-Aime M (2007). Impact ofahospice home visit team onunwanted hospitalization ofterminally-ill patients at home inacute medical emergencies. *Presse Medicale*.

[118] Cinnamon J, Schuurman N, Crooks VA (2008). A method to determine spatial access to specialized palliative care services using GIS. *BMC Health Services Research*.

[119] Guerriere DN, Coyte PC (2011). The ambulatory and home care record: a methodological framework for economic analysies in End-of-Life Care. *Journal of Ageing Research*.

[120] Fealy G, McCarron M, O’Neill D (2009). Effectiveness of gerontologically informed nursing assessment and referral interventions for older persons attending the emergency department: systematic review. *Journal of Advanced Nursing*.

[121] Rodenberg H (2003). Follow the ethical path. *A Journal of Emergency Medical Services*.

[122] Truog RD, Rockoff MA (1991). Ethical issues in pediatric anesthesia. *Seminars in Anesthesia*.

[123] Innes G, Wanger K (1999). Dignified death or legislated resuscitation?. *Canadian Medical Association Journal*.

[124] Schmidt TA, Zechnich AD, Doherty M (1998). Oregon emergency medical technicians’ attitudes toward physician-assisted suicide. *Academic Emergency Medicine*.

[125] Furrow BR (1995). Setting limits in the dying zone: assisted suicide, scarce resources, and hard cases. *University of Detroit Mercy Law Review*.

[126] Peters PG (1998). The illusion of autonomy at the end of life: unconsented life support and the wrongful life analogy. *UCLA Law Review*.

[127] Wessels H, De Graeff A, Wynia K (2010). Are health care professionals able to judge cancer patients’ health care preferences correctly? A cross-sectional study. *BMC Health Services Research*.

[128] Considine J, Miller K (2010). The dialectics of care: communicative choices at the end of life. *Health Communication*.

[129] Marr L, Billings JA, Weissman DL (2007). Spirituality training for palliative care fellows. *Journal of Palliative Medicine*.

[130] Mularski RA, Dy SM, Shugarman LR (2007). A systematic review of measures of end-of-life care and its outcomes. *Health Services Research*.

[131] Pugh EJ, Smith S, Salter P (2010). Offering spiritual support to dying patients and their families through a chaplaincy service. *Nursing Times*.

[132] Thomas K, Lobo B (2011). *Advance Care Planning in End of Life Care*.

[133] Garrett PW, Forero R, Dickson HG, Whelan AK (2008). How are language barriers bridged in acute hospital care? The tale of two methods of data collection. *Australian Health Review*.

[134] Shanley C, Whitmore E (2008). *Preparing for End-of-Life in Residential Aged Care*.

[135] Hancock K, Clayton JM, Parker SM (2007). Truth-telling in discussing prognosis in advanced life-limiting illnesses: a systematic review. *Palliative Medicine*.

[136] ACPEL http://www.acpelsociety.com/index.php.

[137] Stone SC, Abbott J, McClung CD, Colwell CB, Eckstein M, Lowenstein SR (2009). Paramedic knowledge, attitudes, and training in end-of-life care. *Prehospital and Disaster Medicine*.

[138] Van Eijk JTM, De Haan M (1998). Care for the chronically ill: the future role of health care professionals and their patients. *Patient Education and Counseling*.

[139] Wiese CHR, Vagts DA, Kampa U (2011). Palliative care and end-of-life patients in emergency situations. Recommendations on optimization of out-patient care. *Anaesthesist*.

[140] McCallion P, McCarron M (2007). Perspective on quality of life in dementia care. *Intellectual and Developmental Disabilities*.

[141] Clayton JM, Hancock K, Parker S (2008). Sustaining hope when communicating with terminally ill patients and their families: a systematic review. *Psycho-Oncology*.

[142] Masucci L, Guerriere DN, Cheng R, Coyte PC (2010). Determinants of place of death for recipients of home-based palliative care. *Journal of Palliative Care*.

[143] McCallion P, McCarron M, Force LT (2005). A measure of subjective burden for dementia care: the caregiving difficulty scale—intellectual disability. *Journal of Intellectual Disability Research*.

[144] McCarron M, Gill M, Lawlor B, Beagly C (2002). A pilot study of the reliability and validity of the Caregiver Activity Survey—Intellectual Disability (CAS-ID). *Journal of Intellectual Disability Research*.

[145] Tranmer JE, Guerriere DN, Ungar WJ, Coyte PC (2005). Valuing patient and caregiver time: a review of the literature. *PharmacoEconomics*.

[146] Adams J, Bailey DE, Anderson RA, Docherty SL (2011). Nursing roles and strategies in end-of-life decision making in acute care: a systematic review of the literature. *Nursing Research and Practice*.

[147] Eyre S (2010). Supporting informal carers of dying patients: the district nurse’s role. *Nursing Standard*.

[148] Pellett C (2009). Provision of end of life care in the community. *Nursing Standard*.

[149] Katelaris A (2011). Time to rethink end-of-life care. *Medical Journal of Australia*.

[150] Lowthian JA, Jolley DJ, Curtis AJ (2011). The challenges of population ageing: accelerating demand for emergency ambulance services by older patients, 1995–2015. *Medical Journal of Australia*.

[151] Weissman DE (2004). Decision making at a time of crisis near the end of life. *Journal of the American Medical Association*.

[152] Reynolds DF, Garrett CK (1998). Avoiding resuscitation in non-hospital settings: no consent forms. *Bioethics forum*.

[153] Shaughnessy EJ (1990). The “do not resuscitate” order in emergency care: the pre-hospital setting. *New York State Bar Journal*.

[154] Hébert PC (1991). Do not resuscitate orders: considerations for family physicians. *Canadian Family Physician*.

[155] Muller D (2005). Do NOT Resuscitate. A well-orchestrated plan for death ends on a brutal note. *Health Affairs*.

[156] Hillman K (2006). Dying in intensive care. *Care of the Critically Ill*.

[157] Fromme EK, Drach LL, Tolle SW (2005). Men as caregivers at the end of life. *Journal of Palliative Medicine*.

[158] Garling P (2008). *Final Report of the Special Commission of Inquiry into Acute Care Services in NSW Public Hospitals*.

[159] Pinnock H, Kendall M, Murray SA (2011). Living and dying with severe chronic obstructive pulmonary disease: multi-perspective longitudinal qualitative study. *British Medical Journal*.

[160] Snooks H, Wrigley H, George S, Thomas E, Smith H, Glasper A (1998). Appropriateness of use of emergency ambulances. *Journal of Accident and Emergency Medicine*.

[161] Tolle SW, Tilden VR, Drach LL, Fromme EK, Perrin NA, Hedberg K (2004). Characteristics and proportion of dying Oregonians who personally consider physician-assisted suicide. *Journal of Clinical Ethics*.

[162] Valenzuela TD, Copass MK (2001). Clinical research on out-of-hospital emergency care. *New England Journal of Medicine*.

